# The curse of the missing heritability

**DOI:** 10.3389/fgene.2013.00225

**Published:** 2013-11-05

**Authors:** Xia Shen

**Affiliations:** Division of Computational Genetics, Department of Clinical Sciences, Swedish University of Agricultural SciencesUppsala, Sweden

**Keywords:** missing heritability, quantitative trait loci, intercross, linkage analysis, genomic kinship

**Figure 1:**
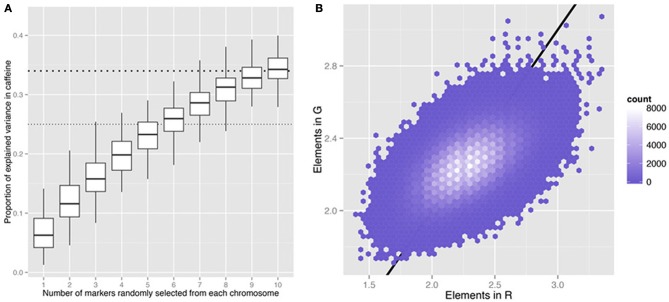
**Information captured by randomly selected markers in the yeast cross^6^. (A)** Proportion of variance explained in the caffeine phenotype by different numbers of randomly selected markers across the genome. Hundred times of random sampling were replicated for each value on the *x*-axis. The thick and thin horizontal dashed lines indicate Bloom et al.'s^6^ estimates of the total narrow sense heritability (*h*^2^) and the *h*^2^ explained by their detected QTL. **(B)** Comparison of the elements in the genomic kinship matrix (G) and those in the kinship matrix estimated by 32 randomly selected markers (R) in the yeast cross. Two markers were randomly selected from each of the 16 yeast chromosomes. *G* = ZZ^*T*^/*n, R* = XX^*T*^/*m*, where *n* is the number of markers across the genome (11,623), *m* is the number of randomly selected markers (32), *Z* is an *N* (number of individuals)-by-*n* matrix of genotype data and *X* is an *N*-by-*m* matrix for the selected markers. The straight line indicates equality and is shown as a visual reference.

Since “the case of the missing heritability” was highlighted 5 years ago (Maher, 2008), scientists have been investigating various possible explanations for this issue (Manolio et al., 2009; Slatkin, 2009; Eichler et al., 2010; Zuk et al., 2012). Recently, Bloom et al. (2013) conducted a linkage analysis in a large yeast *Saccharomyces cerevisiae* cross with high statistical power to map functional quantitative trait loci (QTL) and found that nearly all the additive genetic contribution can be explained by the detected QTL. It is striking that the “old-fashioned” linkage analysis can resolve the missing heritability problem arisen in the high-throughput genome-wide association study (GWAS) era. Compared to human population studies, an intercross creates large linkage disequilibrium (LD) blocks that greatly enhance statistical power but also reduce QTL mapping resolution. Simple simulations (Figure 1) indicate that the real sources or architecture of missing heritability will remain undiscovered due to LD. Breaking down LD would provide better resolution but reduce the power. This commentary is raised to emphasize the trade-off between resolution and statistical power in mapping functional loci.

Linkage analysis or QTL interval mapping in an experimental design is a classic method in quantitative genetics to detect QTL, which allows inferring QTL effects in an un-typed chromosomal interval harbored by flanking genetic markers (Lynch and Walsh, 1998). In an F_2_ cross, the observed LD blocks are often very large, due to limited number of recombination events happened in the F_1_ individuals, though the recombination rate in yeast is relatively high. For example, among the detected QTL for yeast growth in E6 berbamine (Figure 3 in Bloom et al., 2013), the two QTL on chromosome 1 covered the two clear LD blocks (not shown) on the chromosome, and the QTL on chromosome 9 covered most of the chromosome. The finding that the detected QTL can explain almost all the narrow sense heritability (*h*^2^) is expected given that the kinship estimates using only the significant QTL are similar to the genomic kinship. Even a small number of randomly selected markers can resemble the genomic kinship and give similar heritability estimates (Figure 1B), because the number of LD blocks in the entire genome is limited. The prediction of trait values using detected QTL was good according to cross validation, because the specific F_2_ population share similar LD patterns, but such prediction would not perform as superior in another population with different LD pattern. Related empirical evidence can be seen in human height (Makowsky et al., 2011) and marker-assisted selection (Dekkers, 2004), where detected QTL were unsuccessful for out-sample prediction purposes.

If a future generation (e.g., F_8_) with small LD blocks is developed from the F_2_, the statistical power for mapping QTL will decrease. One reason is that a single-locus test for QTL within a large LD block is very likely boosted by multiple QTL within the LD block whose effects are much smaller. The single QTL effect can be simply a combined effect of multiple QTL, and its standard error is underestimated without considering the linkage with other QTL in the same LD region. Assume that there are two functional SNPs *x*_1_ and *x*_2_ in a chromosomal region with high LD, and the phenotype *y* is determined by *y* = *x*_1_β_1_ + *x*_2_ β_2_ + *e* (1), where β_1_ and β_2_ are the effects of the two SNPs; *y, x*_1_, and *x*_2_ are column vectors of data; *e* is a vector of residuals. Due to the high LD, *x*_1_ ≈ *x*_2_ if *x*_1_ and *x*_2_ are on the same scale, so that *y* ≈ *x*_1_(β_1_ + β_2_) + *e*. In a regression model on the single SNP *x*_1_, *y* = *x*_1_β + *e* (2), the estimated effect for β will be approximately β_1_ + β_2_, *i.e*., a combined effect of both variants. Comparing regression models (1) and (2), the standard error (s.e.) of the estimated β is an underestimate of the s.e. of β_1_. This is because the s.e. of β_1_ is inversely proportional to $\sqrt{1-r^{2}}$ where *r* is the correlation coefficient between *x*_1_ and *x*_2_, which is close to 1 due to the high LD, therefore the s.e. of β_1_ becomes much larger than that of β. When the large LD blocks are broken down, such a combined effect will substantially decrease, leading to lack of statistical power for mapping multiple QTL in the original large LD blocks. One previous empirical example was found in chicken advanced intercross lines (AIL), where only five out of nine QTL detected in the F_2_ were confirmed by the AIL (Besnier et al., 2011).

Bloom et al.'s study clearly shows that nearly all the *h*^2^ in yeast is written in the DNA, which improves our understanding of missing heritability though some resolution is sacrificed. Researchers are searching for genetic architecture that answers not only where but also what and how the sources contribute to the heritability. However, the curse of missing heritability forces us to choose between resolution and power. For many complex traits, such as human height (Yang et al., 2011), their polygenic nature makes it extremely difficult to fine-map even the major contribution of the heritability. In future studies, it is important to check the prediction performance in a validation population, in order to show the real sources of missing heritability. Also, biological information and useful tools other than statistical methods need to be developed and utilized.
